# Therapeutics of the Throat: Cistus

**Published:** 1888-01

**Authors:** E. J. Lee


### THERAPEUTICS OF THE THROAT: CISTUS.

THROAT in general. Dryness of throat; feeling of dryness and heat, worse after sleep; better after eating or drinking. Also a small dry spot in gullet (in larynx: *Con.*), which is worse after sleeping; at night must rise and drink; eating relieves this dryness (Berb., Tellur.) even better than drinking. Dryness is worse from midday to midnight. Constantly obliged to swallow to relieve the dryness, especially at night. (Desire to swallow on account of burning in throat: Acon., Bov.)

There is a feeling of coolness in throat; inhaling *cold air causes pain in throat.* There are sensations such as a feeling of sand in throat, or a feeling of softness (both peculiar to this drug).

Of pains, etc., we find: Crawling-itching; periodical itching; tickling and soreness; tearing pain on coughing (All-c., Phos.); rawness extending to chest; in morning sore pain in throat and dryness of the tongue (*Nux mos.*).

Hawking of tough, gum-like mucus, which is tasteless, mostly in the morning. On back part of throat (pharynx) are seen stripes of tough mucus (like Kali c.; Acet-ac., has film of mucus seen on throat). Feels much relieved after getting the phlegm up. *Stitches in the throat, after mental excitement, causing cough.* The fauces inflamed and dry without any dry feeling; throat looks glassy. Externally, there are scrofulous swelling and suppuration of the glands.

LARYNX. Inhaled air feels cool. (Also *Brom.*, Rhus, and Sulph.)

Itching and scratching in larynx, with anxious dreams. Chronic itching. Feeling as if windpipe were contracted. (*Phosp.* suffocative contraction at night on waking.) Pain in trachea. (Under this heading it might be well to quote a peculiar symptom of Cistus, given by Hering: In evening soon after lying down a sensation as if ants were running through whole body; then anxious, difficult breathing; is obliged to get up and open the window; fresh air relieves him; immediately on lying down again these sensations return.

Cough. Mental agitation excites cough; tearing pain in throat from the cough. Expectoration is bitter, and relieves patient very much.

Comments.—Cistus has not very numerous throat symptoms, but most of those here given are peculiar to this remedy. The cough worse from mental excitement, which causes stitches in throat, the feeling of softness, or that as if sand were in throat, the pain from inhaling cold air, are all peculiar to this drug.

Dryness of the throat, etc., is also a prominent symptom; but many drugs have it. Nux mos., mouth tongue and throat are so dry patient can hardly move tongue. Senega, throat so dry it hurts to talk. Dioscorea, throat so dry can't even belch. Sanguinaria, throat so dry feels as if it would crack; also burning, worse after eating sweets. The dryness relieved more by eating than drinking is peculiar and is found also under Berb. and Tellur; while Phell. and Phyt. have it relieved after breakfast.

This cold feeling of Cistus in throat is also found in stomach, before and after eating, in whole abdomen, in the nose, the eructations and saliva and the breath feel cool, finger tips are sensitive to cold air, forehead feels cold externally and internally also. The patient generally is apt to be sensitive to cold air.

Cistus is most suitable to scrofulous patients, with swollen glands, sensitiveness to air, and worse from mental agitation and vexation. Compare with: Arg-n., Calc., Carb-v., Graph., Kali-bi., Kali c., Lach., Nitr-ac., Paris, Phosp., Sulph., etc.

E. J. Lee.

---

